# Combination ergotamine and caffeine improves seated blood pressure and presyncopal symptoms in autonomic failure

**DOI:** 10.3389/fphys.2014.00270

**Published:** 2014-07-24

**Authors:** Amy C. Arnold, Claudia E. Ramirez, Leena Choi, Luis E. Okamoto, Alfredo Gamboa, André Diedrich, Satish R. Raj, David Robertson, Italo Biaggioni, Cyndya A. Shibao

**Affiliations:** ^1^Division of Clinical Pharmacology, Department of Medicine, Autonomic Dysfunction Center, Vanderbilt University School of MedicineNashville, TN, USA; ^2^Department of Biostatistics, Vanderbilt University School of MedicineNashville, TN, USA

**Keywords:** autonomic failure, orthostatic hypotension, ergotamine, caffeine, pressor agent

## Abstract

Severely affected patients with autonomic failure require pressor agents to counteract the blood pressure fall and improve presyncopal symptoms upon standing. Previous studies suggest that combination ergotamine and caffeine may be effective in the treatment of autonomic failure, but the efficacy of this drug has not been evaluated in controlled trials. Therefore, we compared the effects of ergotamine/caffeine on seated blood pressure and orthostatic tolerance and symptoms in 12 primary autonomic failure patients without history of coronary artery disease. Patients were randomized to receive a single oral dose of placebo, midodrine (5–10 mg), or ergotamine and caffeine (1 and 100 mg, respectively) in a single-blind, crossover study. Blood pressure was measured while patients were seated and after standing for up to 10 min, at baseline and at 1 h post-drug. Ergotamine/caffeine increased seated systolic blood pressure (SBP), the primary outcome, compared with placebo (131 ± 19 and 95 ± 12 mmHg, respectively, at 1 h post-drug; *p* = 0.003 for time effect). Midodrine also significantly increased seated SBP (121 ± 19 mmHg at 1 h post-drug; *p* = 0.015 for time effect vs. placebo), but this effect was not different from ergotamine/caffeine (*p* = 0.621). There was no significant effect of either medication on orthostatic tolerance; however, ergotamine/caffeine improved presyncopal symptoms (*p* = 0.034). These findings suggest that combination ergotamine and caffeine elicits a seated pressor response that is similar in magnitude to midodrine, and improves symptoms in autonomic failure. Thus, ergotamine/caffeine could be used as an alternate treatment for autonomic failure, in carefully selected patients without comorbid coronary artery disease.

## Introduction

The autonomic nervous system maintains blood pressure during standing (Smith et al., 1994; Diedrich and Biaggioni, 2004). A failure of this system, as in autonomic failure, results in orthostatic hypotension. Severely affected patients with autonomic failure can only stand for a few seconds to minutes before developing disabling presyncopal symptoms and even syncope, and often exhibit low blood pressure even in the seated position. Although non-pharmacologic measures such as physical counter-maneuvers are the first step in treatment of autonomic failure, pressor agents are often needed in severe patients to adequately control symptoms (Arnold and Shibao, 2013). Currently, there are only two drugs approved for treatment of orthostatic hypotension, the α_1_-adrenergic agonist midodrine and the norepinephrine prodrug droxidopa. The use of these drugs can be limited by adverse effects, however, and the FDA recently proposed the withdrawal of midodrine due to lack of post-marketing evidence to support its clinical efficacy (Dhruva and Redberg, 2010). Thus, there is an emerging need to identify additional therapeutic options for patients with autonomic failure.

Previous small studies, ranging from 1 to 10 patients, have shown that the α-adrenergic agonist ergotamine increases standing blood pressure and improves symptoms in patients with orthostatic hypotension when given by inhaled, intramuscular, or oral routes of administration (Bevegard et al., 1974; Benowitz et al., 1980; Fouad et al., 1981; Chobanian et al., 1983; Hoeldtke et al., 1986; Biaggioni et al., 1990). Oral administration of ergotamine combined with caffeine, to improve its effectiveness and absorption (Schmidt and Fanchamps, 1974), also improved standing blood pressure and presyncopal symptoms in a study of eight autonomic failure patients with parkinsonism and refractory orthostatic hypotension (Dewey et al., 1998). In this previous study, however, patients were administered ergotamine/caffeine in an open-label and uncontrolled fashion. Therefore, the aim of this study was to systematically evaluate the effect of ergotamine/caffeine, compared with placebo and the standard of care midodrine, on seated blood pressure and orthostatic tolerance and symptoms in patients with severe autonomic failure.

## Materials and methods

### Autonomic failure patients

A total of 12 primary autonomic failure patients were recruited from referrals to the Vanderbilt Autonomic Dysfunction Center. Patients had a documented history of neurogenic orthostatic hypotension, defined as ≥20 mmHg decrease in systolic blood pressure (SBP) and/or ≥10 mmHg decrease in diastolic blood pressure (DBP) within 3 min of standing (Freeman et al., 2011), and severe autonomic impairment. The consensus criteria from the American Autonomic Society were used to ascertain the diagnosis of autonomic failure (Freeman et al., 2011). Eight patients were diagnosed with pure autonomic failure (PAF), a primary neurodegenerative disorder characterized by post-ganglionic autonomic denervation and neurogenic orthostatic hypotension, 2 patients with Parkinson's disease (PD+) and 2 with multiple systems atrophy of parkinsonian type (MSA-P). Patients were excluded if they had secondary causes of autonomic failure (e.g., diabetes mellitus, amyloidosis, autoimmune autonomic ganglionopathy) or had current or history of coronary artery disease. All procedures were approved by the Vanderbilt Institutional Review Board and patients provided written informed consent. This study was registered at ClinicalTrials.gov under “Treatment of Orthostatic Hypotension in Autonomic Failure” (NCT00223691).

### General protocol

Patients were admitted to the Vanderbilt General Clinical Research Center, and placed on a low-monoamine, caffeine-free, diet containing 150 mEq sodium and 60–80 mEq potassium per day, for at least 3 days before evaluation. All patients underwent a comprehensive medical history and physical examination. Medications affecting the autonomic nervous system and blood volume (such as fludrocortisone) were withheld for at least five half-lives before admission. Both PD+ patients, however, remained on carbidopa/levodopa (sinemet) for the duration of their inpatient stay due to safety concerns. All patients were evaluated with standardized autonomic function tests (e.g., orthostatic stress, sinus arrhythmia, Valsalva maneuver, hyperventilation, cold pressor, and isometric handgrip tests) (Low, 2003; Okamoto et al., 2012). Orthostatic stress testing was consistently performed starting at 8 a.m. to avoid circadian differences in hemodynamic and biochemical measures. Blood pressure and heart rate were measured after patients were supine for at least 30 min and again after 1, 3, 5, and 10 min of standing, or as long as tolerated, using an automated brachial sphygmomanometer cuff (Dinamap ProCare 100, GE Healthcare). Supine and standing blood samples were collected from an antecubital vein catheter, placed at least 30 min before testing, for measurement of plasma norepinephrine by HPLC with electrochemical detection (Goldstein et al., 1988).

### Acute medication trials

Autonomic failure patients participated in a crossover study with oral placebo, 5 or 10 mg midodrine (Shire PLC) and combination 1 mg ergotamine and 100 mg caffeine (Cafergot, Novartis Pharmaceuticals) administered on separate days. The doses of midodrine and ergotamine/caffeine have been previously shown to elicit pressor responses in autonomic failure patients (Low et al., 1997; Dewey et al., 1998; Wright et al., 1998). Patients were administered the dose of midodrine that they were prescribed to regularly take at home, reflecting standard of care. There were four patients that received the 5 mg dose (2 PAF, 2 MSA) and eight patients that received the 10 mg dose (6 PAF, 2 PD+). The order of medications was randomized using computer generated random numbers, and was blinded to patients (single-blind).

Acute medication trials were conducted at least 2 h after meals to avoid post-prandial hemodynamic effects, and in post-void state. Patients were seated in a chair with their feet on the floor, consistent with our previous trials of pressor agents in autonomic failure (Jordan et al., 1998; Shibao et al., 2010; Okamoto et al., 2012). Blood pressure was measured using an automated brachial sphygmomanometer cuff placed on the right arm, and heart rate by continuous ECG (Dinamap). Data were digitally acquired into a custom database (Microsoft Access, Microsoft Corporation). During baseline, seated blood pressure and heart rate were measured every 5 min for 30 min. Orthostatic tolerance was assessed by measuring blood pressure and heart rate after 1, 3, 5, and 10 min of standing, or as long as tolerated. Patients were then asked to sit and were given the study medication with 50 ml tap water. Seated blood pressure and heart rate were measured every 5 min for 60 min after drug administration, with orthostatic tolerance assessed at the end of this period. We assessed blood pressure at 60 min post-drug as it corresponds to the peak effect of midodrine on standing blood pressure in autonomic failure (Wright et al., 1998), and the time for peak plasma levels of ergotamine (Silberstein and McCrory, 2003). Ergotamine has been shown, however, to improve orthostatic hypotension and symptoms up to 2 h after oral or inhaled administration in autonomic failure (Biaggioni et al., 1990; Dewey et al., 1998). Patients were asked to self-rate the severity of presyncopal symptoms at the end of baseline and post-drug standing periods using a validated orthostatic symptom score (Kaufmann et al., 2012). This score comprises 6 symptoms: (1) dizziness, light-headedness, feeling faint or feeling like you might black out; (2) problems with vision (blurring, seeing spots, tunnel vision); (3) generalized weakness; (4) fatigue; (5) trouble concentrating; and (6) head/neck discomfort. These symptoms are rated individually in terms of severity on a scale of 0 to 10, with 0 reflecting absence of symptoms. Changes in composite symptom scores from baseline to 60 min post-drug were compared for each medication. In addition, changes in scores from Question 1 (lightheadedness), the symptom most commonly described by patients with orthostatic hypotension (Kaufmann et al., 2012; Shibao et al., 2012), were compared for each medication from baseline to 60 min post-drug. All procedures were performed by trained research nurses and in accordance with institutional guidelines.

### Statistical analysis

We tested the null hypothesis that there is no difference in seated blood pressure following placebo, midodrine, and ergotamine/caffeine administration in autonomic failure. The primary outcome was defined *a priori* as the seated SBP during the 60-min post-drug period, with adjustment for average seated SBP at baseline. Baseline and post-drug seated SBP values were logarithmic transformed to reduce skewness in their distribution. A random effects model with random intercept and random slope for time was used to examine for differences in the mean log seated SBP during the 60-min post-drug period among treatment groups. The following terms were included in the model: treatment group, time, time and treatment interaction, and average seated SBP at baseline. Differences in baseline seated SBP among study days were compared with Friedman non-parametric tests. Differences in the seated pressor response between midodrine and ergotamine/caffeine were compared using a McNemar non-parametric test. Secondary outcomes included orthostatic tolerance and presyncopal symptoms measured by the orthostatic symptoms score. Orthostatic tolerance was defined as area under the curve for standing SBP calculated by the trapezoidal rule (AUC_SBP_: upright SBP multiplied by standing time). The difference in the AUC_SBP_ between baseline and post-drug (ΔAUC_SBP_) was used to test whether orthostatic tolerance differed among treatment groups using a random effects model. Comparisons were made only for patients who could stand after all active medications. Differences in orthostatic symptom scores from baseline to 60 min post-administration were compared within each individual drug using Wilcoxon signed-rank non-parametric tests. Data are presented as mean ± 95% confidence interval (CI) unless otherwise specified. All tests were two-tailed, and a *p*-value < 0.05 was considered significant. Analyses were performed with SPSS (version 22.0, IBM Corporation, Chicago, IL) and STATA (version 12.0, StataCorp, College Station, TX).

Sample size calculations were performed using paired *t*-test analysis in PS software (Version 3.0.34) (Dupont and Plummer, 1990). A blinded analysis was performed on preliminary data obtained from the first 3 patients enrolled in this study to obtain an estimate of variance, and showed an approximate 17 mmHg standard deviation of difference in seated SBP among treatment groups. An increase in seated blood pressure of 20 mmHg would be a clinically meaningful difference, representing the approximate magnitude of response achieved with other vasoconstrictor drugs (Jordan et al., 1998). Based on these data, we estimated that 10 patients would have 90% power to detect a difference in means among treatment groups. Since sample size calculations were performed using parametric tests, we increased the sample size by 15% to account for analysis of outcomes with non-parametric methods. Thus, 12 patients were included in this study.

## Results

### Resting cardiovascular autonomic function

Patient clinical characteristics are shown in Table 1. During screening, two patients (1 PAF, 1 PD+) were unable to stand for 1 min during the orthostatic stress testing due to presyncopal symptoms (Table 2). The remaining 10 patients exhibited profound decreases in SBP and DBP after 1 min of standing, without an adequate compensatory increase in heart rate. Autonomic failure patients had severe sympathetic and parasympathetic impairment compared with healthy subjects as indicated in Table 3 by: (a) reduced sinus arrhythmia ratio; (b) reduced Valsalva maneuver heart rate ratio; (c) exaggerated depressor response during phase II and absence of blood pressure overshoot during phase IV of the Valsalva maneuver; and (d) impaired pressor responses to isometric handgrip exercise or pain stimulus (cold pressor test).

**Table 1:** **Patient clinical characteristics**.

**Patient**	**Diagnosis**	**Sex**	**BMI (kg/m^2^)**	**Age (years)**	**Disease duration (years)**
1	PAF	M	25	83	7
2	PAF	M	30	47	6
3	PAF	M	34	65	2
4	PAF	M	29	70	2
5	PD+	F	20	71	10
6	PAF	F	23	70	2
7	MSA-P	M	33	57	2
8	PAF	M	25	69	8
9	PAF	F	26	65	10
10	PAF	M	30	65	6
11	PD+	F	22	56	7
12	MSA-P	M	32	52	4

*BMI, body mass index; PAF, pure autonomic failure; PD+, Parkinson's disease with autonomic failure; MSA-P, multiple systems atrophy with parkinsonian features*.

**Table 2:** **Orthostatic stress testing**.

**Patient**	**Blood pressure (mmHg)**		**Heart rate (bpm)**		**Plasma NE (pg/ml)**	
	**Supine**	**Upright**	**Supine**	**Upright**	**Supine**	**Upright**
1	182/85	114/62	61	68	106	200
2	148/100	70/45	71	81	38	67
3	111/59	66/46	74	111	117	140
4	139/87	65/47	62	86	25	84
5	139/74	88/47	78	98	55	44
6	159/69	66/40	74	103	219	306
7	119/77	83/61	69	81	251	540
8	112/65	66/41	69	75	30	22
9	95/64	- -/- -	93	- -	378	- -
10	127/83	95/61	70	82	122	303
11	86/64	- -/- -	77	- -	274	- -
12	136/87	94/58	85	85	135	184

*Hemodynamic measurements and blood samples were obtained while patients were supine (at least 30 min) and upright (after 1 min of standing). Blood pressure values represent systolic/diastolic. NE, norepinephrine; - -, patient was not able to stand for 1 min to obtain hemodynamic measurements and blood samples due to presyncopal symptoms*.

**Table 3:** **Autonomic function tests**.

**Patient**	**SA ratio**	**Valsalva ratio**	**Valsalva phase II ΔSBP**	**Valsalva phase IV ΔSBP^†^**	**HVTN ΔSBP**	**Cold pressor ΔSBP**	**Handgrip ΔSBP**
1	1.07	1.11	−77	−22	- -	- -	- -
2	1.01	0.95	−62	−50	−42	−10	9
3	1.04	1.07	−88	−42	−37	5	3
4	1.05	1.01	−71	−42	−7	−3	−7
5	1.05	1.06	−29	−60	−26	5	3
6	1.02	1.17	−140	−69	−81	- -	30
7	1.08	1.05	−70	−36	−28	−2	6
8	1.19	1.48	−29	−24	- -	- -	- -
9	1.01	1.03	−146	−97	−53	4	−26
10	1.14	1.13	−38	−23	−10	11	10
11	1.01	1.18	−55	−46	−11	8	2
12	1.00	1.01	−50	−38	−34	3	8
Healthy	1.20 ± 0.1	1.50 ± 0.2	≤20	>20	−5 ± 6	24 ± 13	16 ± 6

*Data for autonomic function testing are reported as changes in systolic blood pressure (ΔSBP) compared with baseline. Data for healthy subjects are presented as mean ± SD and were obtained from the Vanderbilt Autonomic Dysfunction Center database. SA, sinus arrhythmia; HVTN, hyperventilation*.

† *A negative value for phase IV of the Valsalva maneuver indicates that the blood pressure overshoot was absent. - -, patient was not able to tolerate test*.

### Pressor response to drugs

**Figure 1:**
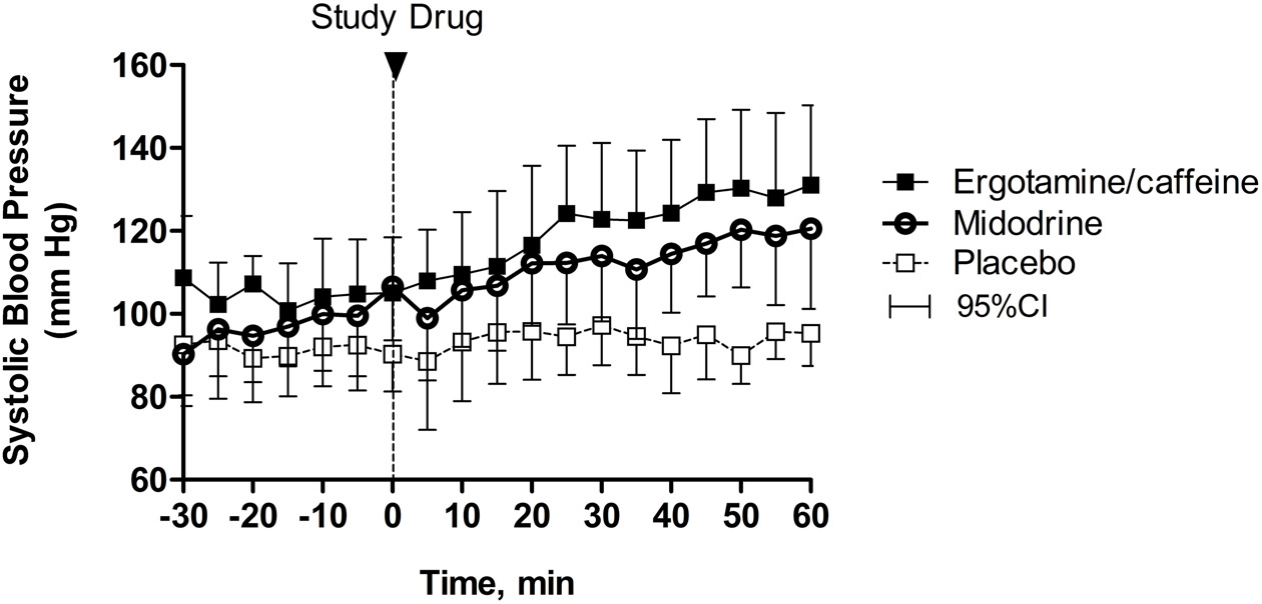
**Changes in seated blood pressure following drug administration**. The combination of 1 mg ergotamine and 100 mg caffeine increased seated systolic blood pressure over time compared with placebo in patients with severe autonomic failure (*p* = 0.003). Similarly, 5–10 mg midodrine increased seated systolic blood pressure in these patients (*p* = 0.015), but this effect was not different from ergotamine/caffeine (*p* = 0.621). Data are presented as mean ± 95% confidence interval.

The average seated SBP during the 30-min baseline period was similar among placebo, ergotamine/caffeine, and midodrine study days (*p* = 0.441; Friedman test). Ergotamine/caffeine significantly increased seated SBP, the primary outcome, as a linear function of time compared with placebo (Figure 1; slope difference: 1.003; 95% CI: 1.001 to 1.005; *p* = 0.003; random effects model). Similarly, midodrine increased seated SBP compared with placebo (Figure 1; slope difference: 1.002; 95% CI: 1.001–1.004; *p* = 0.015; random effects model). The magnitude of the seated pressor response to midodrine was similar between patients receiving the 5 mg (median: 26; IQR: 14–36) and 10 mg (median: 23; IQR: 7–48) doses. There was no significant difference, however, in the seated pressor response between ergotamine/caffeine and midodrine (slope difference: 1.000; 95% CI: 0.998–1.001; *p* = 0.621; random effects model). In this study, 9 out of 12 (75%) patients exhibited an increase in seated SBP ≥ 20 mmHg with ergotamine/caffeine compared with 5 out of 12 (42%) patients for midodrine, a difference that was not significantly different (*p* = 0.125; McNemar test).

### Orthostatic tolerance and symptoms

**Figure 2:**
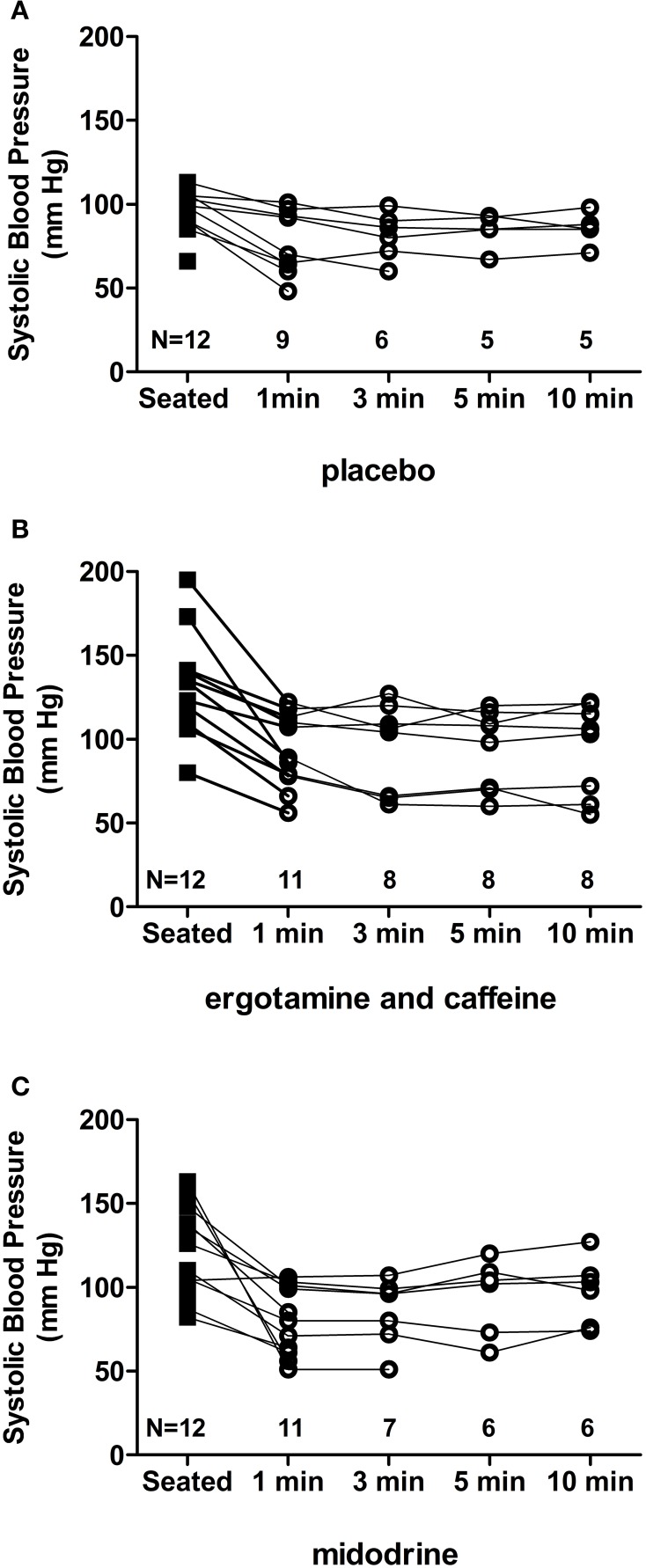
**Orthostatic tolerance following drug administration**. At 60 min following drug administration, autonomic failure patients were asked to stand for 10 min, or as long as tolerated. Systolic blood pressure was measured at 1, 3, 5, and 10 min to assess orthostatic tolerance. Blood pressure data and standing time are shown for each individual patient, with *N* representing the number of patients who could tolerate standing at a given time point. Five out of 12 (42%) patients could stand for 10 min following placebo **(A)** compared with 8 out of 12 (67%) after ergotamine/caffeine **(B)** and 6 out of 12 (50%) after midodrine **(C)** administration.

**Figure 3:**
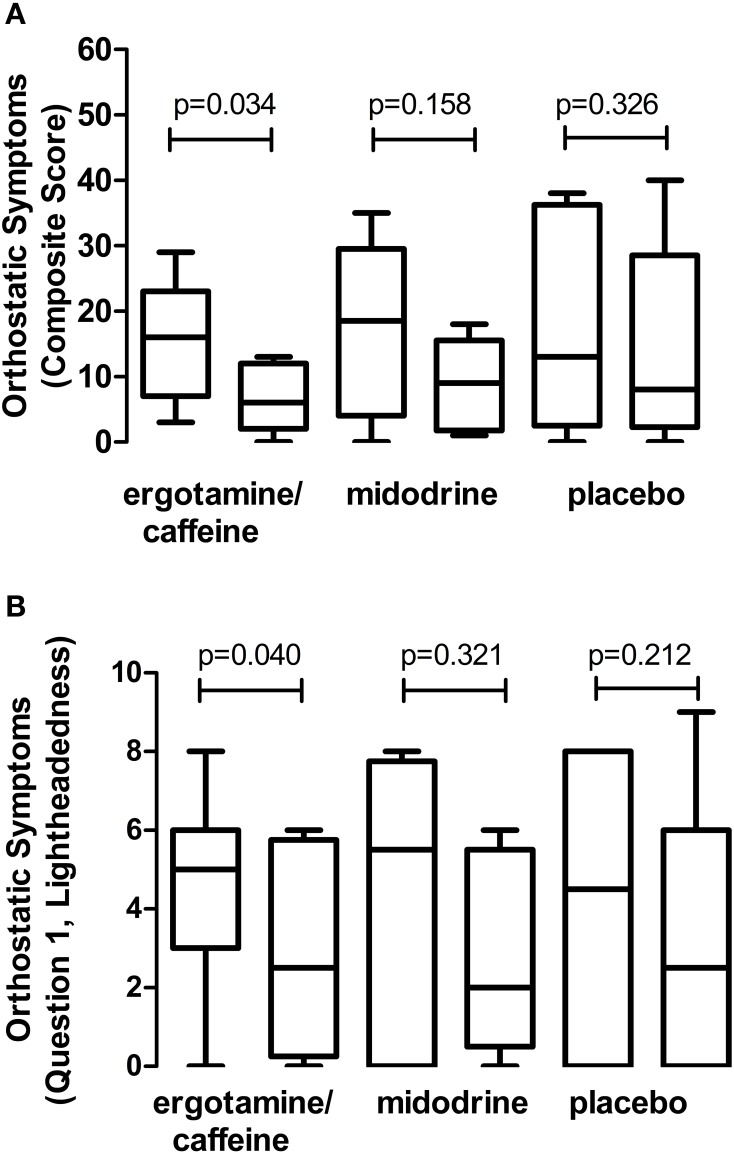
**Effect of drugs on orthostatic presyncopal symptoms**. Data represent changes in orthostatic symptom scores from baseline (left bar) to 60 min post-administration (right bar), with comparisons made for each individual drug. **(A)** Combination 1 mg ergotamine and 100 mg caffeine reduced the orthostatic symptom composite score in autonomic failure patients, suggesting an improvement in presyncopal symptom burden. In contrast, there was no effect of 5–10 mg midodrine or placebo on this composite score. **(B)** Combination ergotamine and caffeine also reduced the lightheadedness component (Question 1) of the orthostatic symptoms score in these patients, with no significant effect of midodrine or placebo. Data are presented as mean ± 95% confidence interval.

As shown in Figure 2, at 60 min after placebo administration 5 out of 12 (42%) autonomic failure patients could stand for 10 min, compared with 8 out of 12 (67%) after ergotamine/caffeine and 6 out of 12 (50%) after midodrine. There was no significant difference, however, in orthostatic tolerance between ergotamine/caffeine and placebo (ΔAUC_SBP_: 248; 95% CI: −73 to 568; *p* = 0.130; random effects model), between midodrine and placebo (ΔAUC_SBP_: 85; 95% CI: −141 to 311; *p* = 0.461), or between ergotamine/caffeine and midodrine (ΔAUC_SBP_: −163; 95% CI: −387 to 62; *p* = 0.155). The orthostatic symptom composite score, as well as the lightheadedness component of this score (Question 1), were reduced at 60 min following ergotamine/caffeine administration (Figure 3; *p* = 0.034 and 0.040, respectively; Wilcoxon signed-rank test) when compared with baseline. In contrast, there was no significant effect of either midodrine or placebo on orthostatic symptom scores (Figure 3).

## Discussion

The main finding of this study is that combination ergotamine and caffeine improves seated blood pressure in patients with severe autonomic failure, to a similar extent as the standard of care pressor agent midodrine. Furthermore, while there was no significant effect of either drug on orthostatic tolerance, ergotamine/caffeine improved presyncopal symptoms upon standing. These findings suggest that the combination of ergotamine and caffeine may be useful for the treatment of autonomic failure, particularly in patients without underlying vascular disease and who are unable to tolerate midodrine.

Autonomic failure is associated with sympathetic denervation and impaired baroreflex-mediated arteriolar vasoconstriction, leading to excessive venous pooling and reduced venous return to the heart upon assumption of the upright position. The peripheral α_1_-adrenergic agonist midodrine increases systemic vascular resistance, and has demonstrated efficacy to elevate blood pressure and improve presyncopal symptoms in autonomic failure (Schirger et al., 1981; Kaufmann et al., 1988; Jankovic et al., 1993; Fouad-Tarazi et al., 1995; Low et al., 1997; Wright et al., 1998). The increase in seated SBP with midodrine was similar in magnitude to a previous report in seated patients (Jordan et al., 1998), and to the effects of chronic administration on standing blood pressure (Jankovic et al., 1993). The use of midodrine is limited in some patients by adverse effects such as pilomotor reactions, pruritus of the scalp, urinary urgency or retention, and supine hypertension (Fouad-Tarazi et al., 1995; Low et al., 1997). We also recently reported poor persistence on treatment with midodrine in patients with neurogenic orthostatic hypotension (Shibao et al., 2013a). Furthermore, not all patients respond to midodrine, with a previous report showing a pressor effect in 69% and 38% of PD+ and MSA patients, respectively (Jankovic et al., 1993). Similarly, in this study only 42% of autonomic failure patients had a significant pressor response to midodrine. These findings illustrate the need to identify additional therapeutic options for the treatment of autonomic failure.

In addition to potent vasoconstriction, ergotamine elicits peripheral venoconstriction through stimulation of α-adrenoreceptors (Fouad et al., 1981; Chobanian et al., 1983). Ergotamine/caffeine could be more effective than midodrine in this regard by improving venous return, but this was not observed. There was a high response rate to ergotamine/caffeine with 75% of patients in this study and 100% of patients in a previous study (Dewey et al., 1998) exhibiting a greater than 20 mmHg increase in seated and standing blood pressure, respectively. Some long-term benefit of ergotamine/caffeine to reduce presyncopal symptoms and syncopal episodes has also been demonstrated in small, uncontrolled studies (Chobanian et al., 1983; Dewey et al., 1998). It is difficult to predict which patients will respond to ergotamine/caffeine, however, as an acute test dose did not predict long-term efficacy in autonomic failure (Dewey et al., 1998). Furthermore, the severity of autonomic failure, results of autonomic function testing, and plasma catecholamine levels were shown to be poor predictors of response to pressor agents (Jordan et al., 1998). The magnitude of pressor responses to midodrine and ergotamine/caffeine (26 and 36 mmHg, respectively) likely reflects loss of baroreflex buffering, but may also involve enhanced adrenergic sensitivity. It is unlikely that caffeine played a major role in the seated pressor effect of ergotamine/caffeine treatment, as acute caffeine produces a modest pressor or no effect in autonomic failure (Onrot et al., 1985; Jordan et al., 1998).

The goal for treatment of autonomic failure is to reduce presyncopal symptoms and improve standing time, and not to treat underlying disease. This can be achieved by increasing seated blood pressure within the range of cerebral autoregulation, so that cerebral perfusion is maintained even with a fall in blood pressure during standing. In this study, orthostatic tolerance was defined as the AUC for standing SBP, to take into account not only blood pressure levels but also total standing time, similar to our previous studies (Shibao et al., 2010; Okamoto et al., 2012). Midodrine and ergotamine have both been shown to improve standing time and presyncopal symptoms in autonomic failure (Chobanian et al., 1983; Kaufmann et al., 1988; Biaggioni et al., 1990; Jankovic et al., 1993; Fouad-Tarazi et al., 1995; Low et al., 1997; Dewey et al., 1998; Wright et al., 1998), perhaps suggesting that this study was underpowered to detect differences in measures of orthostatic tolerance.

In this study, five patients were discharged on ergotamine/caffeine, of which three continued taking this medication in combination with other pressor agents (e.g., midodrine, pyridostigmine). Two patients subsequently stopped taking ergotamine/caffeine, one due to side effects (feeling tense) and the other because the medication was not readily available. A previous study also reported nausea and tachyphylaxis with chronic administration in autonomic failure (Dewey et al., 1998). Importantly, ergotamine can produce vascular complications such as arterial vasospasm (ergotism) and valvular heart disease. There was no evidence of vascular complications with chronic ergotamine/caffeine in autonomic failure (Dewey et al., 1998), even at doses reported to cause ergotism in migraine patients (Bigal and Tepper, 2003). There is limited data, however, on long-term safety and efficacy for treatment of orthostatic hypotension. Thus, this drug is recommended only in carefully selected patients without evidence of coronary or peripheral artery disease.

At least half of autonomic failure patients also have paradoxic supine hypertension (Biaggioni and Robertson, 2002). The ideal pressor agent in these patients would prevent the fall in blood pressure with standing, without increasing supine or seated blood pressure. There have been no drugs to date, however, that have met this criteria. Initial studies showed that ergotamine preferentially increases standing blood pressure in patients with orthostatic hypotension (Jennings et al., 1979; Olver et al., 1980), but this has not been a consistent finding with reports of limiting supine hypertension (Chobanian et al., 1983; Dewey et al., 1998). We did not measure supine blood pressure in this study for safety reasons, but similar to other short-acting pressor agents, the use of ergotamine/caffeine should be limited to morning and early afternoon with avoidance of the supine position for at least 4 h after each dose, following recent standard of care guidelines (Shibao et al., 2013b).

There are some potential limitations to this study. First, autonomic failure patients were enrolled at a tertiary care center for autonomic disorders and may not reflect the broader and less severe disease population. Second, this study included a relatively small number of patients, but was similar in sample size to previous studies of pressor agents in autonomic failure. This study was powered to detect an increase in seated SBP, the primary outcome; however, secondary outcomes including orthostatic tolerance may have reached significance with additional patients. Third, we were not able to evaluate standing blood pressure as our primary endpoint, as most patients are only able to stand for a few seconds to minutes due to disabling presyncopal symptoms. The seated position provides a well-tolerated postural stress in these patients that allows for evaluation over prolonged time periods, and in most patients we have found that the acute seated pressor response is a reasonable predictor of long-term symptomatic efficacy.

## Author contributions

All authors have given final approval of this version of this manuscript, and have agreed to be accountable for all aspects of this work. The following additional author contributions are acknowledged: study conception and design (Luis E. Okamoto, Alfredo Gamboa, André Diedrich, Satish R. Raj, David Robertson, Italo Biaggioni, and Cyndya A. Shibao), acquisition, analysis or interpretation of data (all authors), drafting of the manuscript (Amy C. Arnold and Cyndya A. Shibao), critical revision of the manuscript for intellectual content (Claudia E. Ramirez, Luis E. Okamoto, Alfredo Gamboa, André Diedrich, Satish R. Raj, David Robertson, Italo Biaggioni, and Leena Choi), obtaining funding (David Robertson and Italo Biaggioni), study supervision (David Robertson, Italo Biaggioni, and Cyndya A. Shibao) and statistical analysis (Leena Choi).

### Conflict of interest statement

Amy C. Arnold was supported by American Heart Association grant 11POST7330010. Claudia E. Ramirez reports no disclosures. Leena Choi reports no disclosures. Luis E. Okamoto reports no disclosures. Alfredo Gamboa is supported by NIH grant K23HL95905. André Diedrich reports no disclosures. Italo Biaggioni is a consultant for Chelsea Therapeutics and Astra Zeneca, is funded by NIH grants P01 HL056693 and U54NS065736, and receives research support from Astra Zeneca and Forest Laboratories. David Robertson is a consultant for Chelsea Therapeutics, and is funded by NIH grants P01HL056693.
